# Assay conditions for estimating differences in base excision repair activity with Fpg-modified comet assay

**DOI:** 10.1007/s10565-023-09801-0

**Published:** 2023-03-17

**Authors:** Congying Zheng, Andrew Collins, Gunnar Brunborg, Frederik-Jan van Schooten, Anne Lene Nordengen, Sergey Shaposhnikov, Roger Godschalk

**Affiliations:** 1 Department of Pharmacology and Toxicology, NUTRIM School of Nutrition and Translational Research in Metabolism, Maastricht University, 6200 Maastricht, Netherlands; 2 Norgenotech AS, 64/66 Ullernchassern, Oslo, Norway; 3 Oslo Cancer Cluster, 64/66 Ullernchassern, Oslo, Norway; 4 Department of Public Health, Sport and Nutrition, University of Agder, 4604 Kristiansand, Norway; 5 Department of Nutrition, University of Oslo, 0372 Oslo, Norway

**Keywords:** Cellular repair, Fpg, Comet assay, DNA repair capacity, Antioxidant status

## Abstract

DNA repair is an essential agent in cancer development, progression, prognosis, and response to therapy. We have adapted a cellular repair assay based on the formamidopyrimidine DNA glycosylase (Fpg)-modified comet assay to assess DNA repair kinetics. The removal of oxidized nucleobases over time (0–480 min) was analyzed in peripheral blood mononuclear cells (PBMCs) and 8 cell lines. DNA damage was induced by exposure to either Ro19-8022 plus visible light or potassium bromate (KBrO_3_). The initial amount of damage induced by Ro 19–8022 plus light varied between cell lines, and this was apparently associated with the rate of repair. However, the amount of DNA damage induced by KBrO_3_ varied less between cell types, so we used this agent to study the kinetics of DNA repair. We found an early phase of ca. 60 min with fast removal of Fpg-sensitive sites, followed by slower removal over the following 7 h. In conclusion, adjusting the initial damage at T_0_ to an equal level can be achieved by the use of KBrO_3_, which allows for accurate analysis of subsequent cellular DNA repair kinetics in the first hour after exposure.

**Graphical Abstract:**

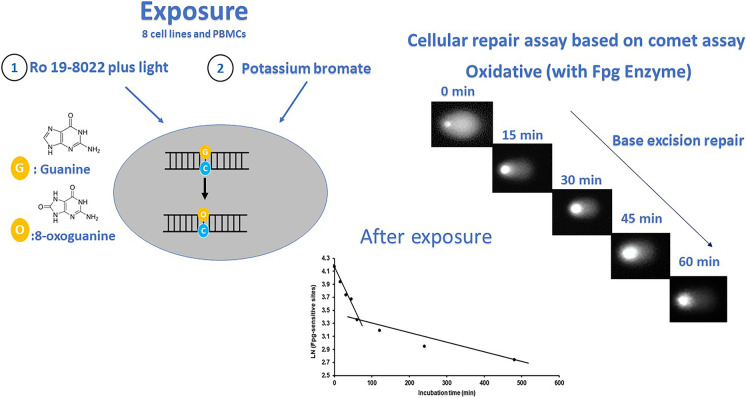

**Supplementary Information:**

The online version contains supplementary material available at 10.1007/s10565-023-09801-0.

## Introduction

Humans are constantly exposed to environmental and occupational hazards that can cause DNA damage. This damaged DNA is removed by DNA repair mechanisms, thus maintaining genome integrity and preventing mutations. Therefore, DNA repair capacity may be a useful biomarker when studying cancer development, progression, prognosis, and response to clinical therapy. To assess inter-individual differences in DNA repair activity, the comet assay (single-cell gel electrophoresis) is a suitable, robust, and sensitive method. Ostling and Johanson as well as Singh et al., in the original papers describing the comet assay for DNA breaks, also applied it to DNA repair, studying the decrease of ionizing radiation-induced strand breaks over time (Ostling and Johanson 1984; Singh et al. 1988). A similar approach can be used for other types of DNA damage that can be detected by the comet assay. The standard comet assay was modified to reveal oxidized bases by incubating the nucleoids after lysis with the bacterial enzyme endonuclease III (Endo III) to convert oxidized pyrimidines to breaks(Collins et al. 1993), or with formamidopyrimidine DNA glycosylase (Fpg) to detect oxidized purines (Dusinska and Collins 1996). Using H_2_O_2_ to induce DNA damage in HeLa cells and human lymphocytes, removal of strand breaks and oxidized bases was followed over time; differences in DNA repair kinetics were found between the human cell line HeLa and freshly isolated human peripheral blood mononuclear cells (PBMCs) (Collins et al. 1995).

The comet assay has already been applied in clinical settings, for example, in studying radiotherapy susceptibility and individualized health risk assessment. Palyvoda et al. (2003) estimated the cellular repair rates of healthy donors and of patients with squamous cell carcinoma of the head and neck (SCCHN) before radiotherapy, by measuring residual strand breaks at six time intervals from 0 min to 3 h after treating lymphocytes with γ-irridation (3 Gy dose) on ice, in order to predict treatment outcome. Ocolotobiche et al. (2021) used this assay to identify patients at higher risk of radiotherapy side effects, although they found no significant difference between patients and healthy volunteers. Valdiglesias et al. (2020) evaluated and compared the suitability of different blood preparations (frozen PBMCs, fresh blood, and frozen blood) isolated from a healthy population and treated with different challenging agents (bleomycin, methyl methane sulfonate (MMS), and UV light) in the cellular repair assay to assess the response to DNA damage. Various DNA-damaging agents can be used to induce different types of lesions in DNA for studying DNA repair activity with the comet assay, namely, photosensitizer Ro 19–8022 plus visible light, potassium bromate (KBrO_3_), MMS, UVC, and benzo(a)pyrene diol epoxide (BPDE). The DNA of cells treated with Ro 19–8022 plus light or KBrO_3_ contains oxidized purines, mainly 8-oxoguanine (8-oxoG), and MMS induces DNA alkylations, substrates for base excision repair (BER). UVC and BPDE induce pyrimidine dimers and bulky adducts, respectively, and are repaired by nucleotide excision repair (NER) (Collins et al. 2002; Speit et al. 2004; Ballmaier and Epe 2006; Luan et al. 2007; Camenisch and Naegeli 2009; Hašplová et al. 2012; Goto et al. 2015). In the current study, we focused on cellular repair after a challenge with Ro 19–8022 plus light, or KBrO_3_. Until now, there have been few studies measuring DNA repair after KBrO_3_ exposure (Kumar et al. 2022; Parlanti et al. 2012; Platel et al. 2011); in these papers, KBrO_3_ was used to induce 8-oxoG in DNA repair gene-deficient or silenced cell lines. Although the mechanism of KBrO_3_-induced DNA damage is not yet fully understood, it seems to provide a useful alternative approach to studying BER of oxidized DNA.

The cellular repair assay can accurately monitor repair kinetics, but some aspects of the assay need to be standardized before it can be used in clinical settings or human biomonitoring, For instance, it is not known whether the level of initial DNA damage affects the rate of repair. It is important to distinguish between the prevention of damage by antioxidant defenses and the removal of damage by DNA repair (Collins and Azqueta 2012; Collins 2014). Our aim in this paper was to identify factors affecting the rate of repair of oxidized bases and to propose a reliable approach to the comparative study of DNA repair rates in different cell lines that could also be of use in studying inter-individual variations in DNA repair in humans.

## Materials and methods

### Chemicals

RMPI-1640 medium, fetal bovine serum, 100 U/ml penicillin, 100 µg/ml streptomycin, trypsin–EDTA 1 × , SYBR Gold, and chemicals and reagents used for the comet assay were purchased from Sigma Aldrich (Heidelberg, Germany); McCoy’s 5 a Medium was purchased from Cytiva AS; Eagle’s Minimum Essential Medium and Dulbecco’s Modified Eagle Medium (DMEM) were purchased from Biowest AS. Trypan blue solution (0.4%) was purchased from Invitrogen Company (Thermo Fisher Scientific). Lymphoprep was purchased from Fresenius Kabi Norge As.

### Cells

The following cell lines were used: HCT-116 (human colorectal carcinoma cell line), LNCaP (human prostate adenocarcinoma cell line), TK-6 (human lymphoblastoid cell line), LLC-pk1 (porcine kidney cell), V79-4 (Chinese hamster lung fibroblast cell line), MCF-7 (human breast cancer cell line), Caco2 (human colorectal adenocarcinoma cell line), HeLa cells (human cervical cell line), and HepG2 (human hepatocellular carcinoma cell line). All cell lines were grown in the appropriate medium, according to the protocol provided by the ATCC; they were incubated at 37 °C in a humidified incubator with a 5% CO_2_ atmosphere.

PBMCs were collected from 5 non-smoking, healthy volunteers (25–35 years) under the approval of the Regional Ethical Committee Southeast Norway. The venous blood (sampled in a vacutainer tube with EDTA as anticoagulant) was diluted in a 15 ml plastic tube at a volume ratio of 1:1 with sterile PBS, underlayed with the same volume of Lymphoprep, and centrifuged at 250 × g for 30 min at 4 °C with the brake off. PBMCs were isolated from the interface between PBS and Lymphoprep, washed with PBS, centrifuged (250 × g, 5 min at 4 °C), and resuspended in 1 ml sterile PBS.

### *Exposure of cells to Ro 19–8022, or KBrO*_*3*_

Non-adherent cells (PBMCs and TK-6) were suspended in RMPI-1640 with 10% fetal bovine serum and placed into Petri dishes at a concentration of 2.5 × 10^5^ cells/ml. Adherent cells were seeded into 24-well plates and allowed to grow to 70–85% confluence and were then detached with 1 × trypsin–EDTA, washed with PBS, centrifuged (250 × g, 5 min at 4 °C), and resuspended in 1 ml sterile PBS. Five hundred microliters of cell suspension was kept as control, and the remaining cells (in a Petri dish) were placed on ice, treated with 1 µM Ro 19–8022 (a gift from F. Hoffmann-La Roche), and irradiated with visible light (33 cm from a 500 W tungsten halogen source) for 5 min.

In later experiments, non-adherent cells were exposed to a range of concentrations of KBrO_3_ between 0 and 100 mM, in appropriate cell medium for 1 h at 37 °C. After treatment, cells were washed with PBS, centrifuged (250 × g, 5 min at 4 °C), and resuspended in 1 ml sterile PBS. Adherent cells were seeded into 24-well plates and allowed to grow to 70–85% confluence, exposed to the same range of concentrations of KBrO_3_ as suspension cells in appropriate cell medium for 1 h at 37 °C after treatment, centrifuged, and resuspended in 1 ml sterile PBS.

### Cytotoxicity test

To assess the Ro 19–8022 plus light or KBrO_3_-induced cytotoxicity, Trypan blue tests were conducted in parallel with the comet assay before and after exposure. In all cases, viability was higher than 80% (Table S1).

### Alkaline comet assay for strand breaks and alkali-labile sites

After treatment with Ro 19–8022 plus light or KBrO_3_, cell suspensions were mixed with 0.7% low melting point agarose (LMPA), and 50 µL was placed on a glass slide pre-coated with 1% normal melting agarose. Gels were set at 4 °C, and the embedded cells were lysed at 4 °C overnight (lysis buffer: 2.5 M NaCl, 100 mM Na_2_EDTA, 10 mM Tris base, pH = 10, and 1% Triton X-100 added just before use). Slides were then placed in an alkaline electrophoresis solution (0.3 M NaOH, 1 mM Na_2_EDTA, pH > 12) for 20 min at 4 °C for unwinding and electrophoresed in the same solution for 20 min at a voltage gradient of 0.8 V/cm across the platform in a horizontal electrophoresis chamber (Bio-Rad, Richmond, CA, USA). Finally, the slides were rinsed once with PBS (1 × , pH = 7.4), twice in distilled water, and left to dry. For scoring, slides were stained with 1 µM SYBR™ Gold at the recommended 10,000 × dilution for 30 min in the dark and then rinsed twice in distilled water.

The Comet IV semi-automated image analysis system was used to evaluate 50 comets per gel. The percentage of DNA in the tail (% tail DNA) was the descriptor used, and the median value of % tail DNA from 100 comets was used to measure DNA damage for each condition.

### Fpg-modified comet assay

Fpg was produced by Norgenotech AS, Norway, and was the same enzyme (made in one batch) as used by the European Comet Assay Validation Group (ECVAG) (Møller et al. 2010). Aliquots were diluted tenfold with Fpg reaction buffer (40 mM HEPES, 0.1 M KCl, 0.5 mM Na_2_EDTA, 0.2 mg/mL BSA, pH = 8) with the addition of 10% glycerol and stored at − 80 °C. For each experiment, an aliquot was diluted with 30 ml of Fpg reaction buffer, reaching a final dilution of 60,000 times from the original crude preparation (0.5 µg/ml total protein). The slides were then placed on a plastic rack, 50 µl Fpg solution or the reaction buffer was added to each gel, and a 22 × 22 mm coverslip was placed on top. Then, the rack was transferred to a pre-heated moist box and placed in an incubator for 1 h at 37 °C. After incubation, the slides were placed at 4 °C in a cold room to stop the Fpg reaction. The coverslips were removed, and all slides were transferred to the electrophoresis tank; subsequent steps were as for the standard comet assay for strand breaks. Net Fpg-sensitive sites were estimated by subtracting % tail DNA with buffer incubation only from % tail DNA with Fpg incubation.

### Cellular repair assay

After treatment with Ro 19–8022 plus light or KBrO_3_, suspension cells were placed in a T75 flask with cell-specific medium at 37 °C in a humidified incubator with a 5% CO_2_ atmosphere to allow for DNA repair. Cells were sampled at 0, 15, 30, 45, 60, and 120 min and collected by centrifugation (250 × g, 5 min at 4 °C). Adherent cells were detached by 1 × trypsin–EDTA at 0, 15, 30, 45, 60, and 120 min after exposure, and collected by centrifugation (250 × g, 5 min at 4 °C). (Additional time points were included for incubations with KBrO_3_, namely, 240 and 480 min.) Cells were resuspended in 1 ml sterile PBS mixed with 0.7% LMPA to prepare gels and analyzed with the comet assay as described.

### *H*_*2*_*O*_*2*_* resistance assay*

To assess antioxidant status, cells were exposed to H_2_O_2_ (0 µM, 12.5 µM, 25 µM, 50 µM, 100 µM) on ice for 5 min and subsequently washed and resuspended in sterile PBS. Cell suspensions were mixed with 0.7% LMPA to prepare gels, which were then processed with the comet assay for strand breaks. This approach of measuring H_2_O_2_ resistance has shown meaningful variation among samples of lymphocytes from individuals, with lower resistance in smokers, and increased resistance after taking a vitamin C supplement (Panayiotidis and Collins 1997).

### Data analysis

Statistical analysis was conducted using IBM SPSS Statistics 21.0 and Excel. The normality of data distribution of all parameters was tested by the Kolmogorov–Smirnov test, then using ANOVA with Dunnett’s post hoc test for differences between groups, and Student’s *t*-test was used for differences between interval 0–60 min and interval 60–480 min. In case the data did not fit a normal distribution, the Kruskal–Wallis test and Mann–Whitney U test were used. All statistical tests were conducted with the confidence level set at 95% (*P* = 0.05). To study the rate of DNA repair, the half-life of DNA damage was estimated assuming first-order kinetics using the formula: ${t}_{(\frac{1}{2})}=\left(-Ln\left(2\right)*t\right)/(Ln\left(\frac{{N}_{t}}{{N}_{0}}\right))$ (*t* = time; *N*_*t*_ = damage at time point t; *N*_0_ = damage at start of measurement) (Petrucci et al. 1997). 

## Results

### Removal of DNA damage after treatment of cells with Ro 19–8022 plus light

**Fig. 1:**
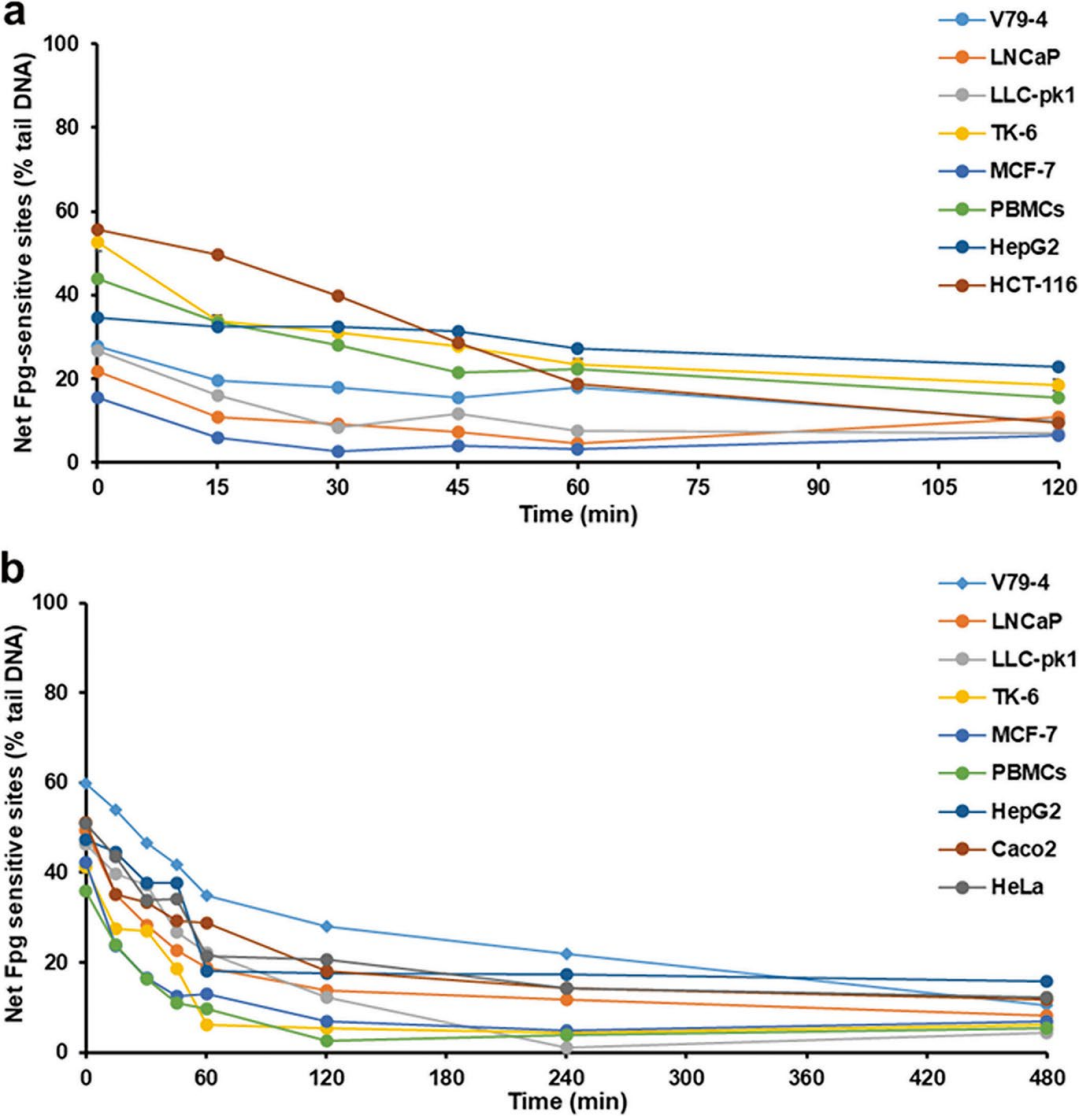
Removal of DNA damage in eight cell lines plus PBMCs treated with **a** 1 µM Ro 19–8022 plus light for 5 min or **b** 10 mM KBrO_3_ for 1 h. After treatment, the cells were incubated to allow repair of the damage in appropriate culture medium at 37 °C for time intervals as indicated. The removal of oxidized bases was monitored using the Fpg-modified comet assay. Data are shown as the mean of median values of three repeat experiments. In the case of PBMCs, there were 2 repeat experiments with each of the 5 samples

**Fig. 2:**
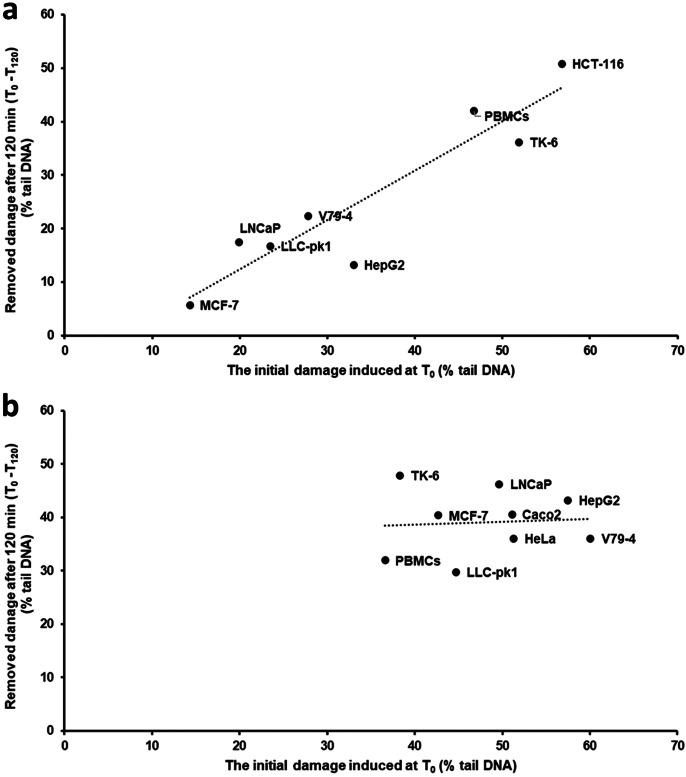
**A** Association between relative decrease of net Fpg-sensitive sites induced by Ro 19–8022 plus 5 min light over the first 120 min of incubation and initial level of net Fpg-sensitive sites induced by this treatment. **b** Association between relative decrease of net Fpg-sensitive sites induced by KBrO_3_ over the first 120 min of incubation and initial level of net Fpg-sensitive sites induced by KBrO_3_

Cell lines as well as PBMCs from healthy volunteers were treated with 1 µM Ro 19–8022 plus light to introduce DNA base oxidation damage, after which the cells were monitored for the removal of DNA damage (i.e., DNA repair) at several time intervals between 0 and 120 min (Fig. 1a). Removal of net Fpg-sensitive sites was seen in all cell lines except MCF-7 and HepG2. It is notable that the initial damage (Fpg-sensitive sites) varied widely between cell lines, ranging between 19.8% tail DNA in MCF-7 and 69.3% tail DNA in HCT-116. We reason that it is important to start with the same level of DNA damage to make a valid comparison between cells, because as shown in Fig. 2a, the DNA damage removal over a period of 120 min was significantly related to the initial DNA damage (*R*^2^ = 0.86, *P* < 0.001), consistent with the law of mass action. Significant levels of removal were found in most of the cell lines, V79-4 (15 min), LLC-pk1, LNCaP (30 min), PBMCs (45 min), HCT-116, TK-6 (60 min); no significant removal was observed in MCF-7 and HepG2.

### *Kinetics of removal of DNA damage after treatment of cells with Ro 19–8022* + *light or with KBrO*_*3*_

Although photosensitizer Ro 19–8022 is frequently used to induce oxidized bases in DNA, finding a suitable concentration of this chemical to produce the same initial DNA damage in all cell lines was a challenge; for instance, doubling the concentration of Ro 19–8022 applied to MCF-7 and V79-4 cells (both cell lines with low initial levels of DNA damage) did not significantly increase DNA damage levels.

We therefore tested KBrO_3_ as an alternative DNA-damaging chemical, which produces more consistent levels of DNA damage (data not shown). Similar levels of damage (around 60–70% tail DNA) were induced by 10 mM KBrO_3_ in the 8 cell types (PBMCs being the exception, with only 35.8% net Fpg-sensitive sites at time zero). The removal of damage (i.e. DNA repair) was monitored for 480 min (Fig. 1b). Some cell lines were able to efficiently repair the KBrO_3_-induced DNA damage reaching background levels already after 120 min, whereas in other cell lines, DNA damage was still significantly higher in the treated cells after 120 min when compared to background levels (Fig. S1) in that same cell type. The percentage removal of damage after 120 min showed no significant correlation with the initial DNA damage induced (*R*^2^ = 0.005, *P* > 0.05) (Fig. 2b).

### *Initial DNA damage (net Fpg-sensitive sites) related to H*_*2*_*O*_*2*_* sensitivity of cells*

**Fig. 3:**
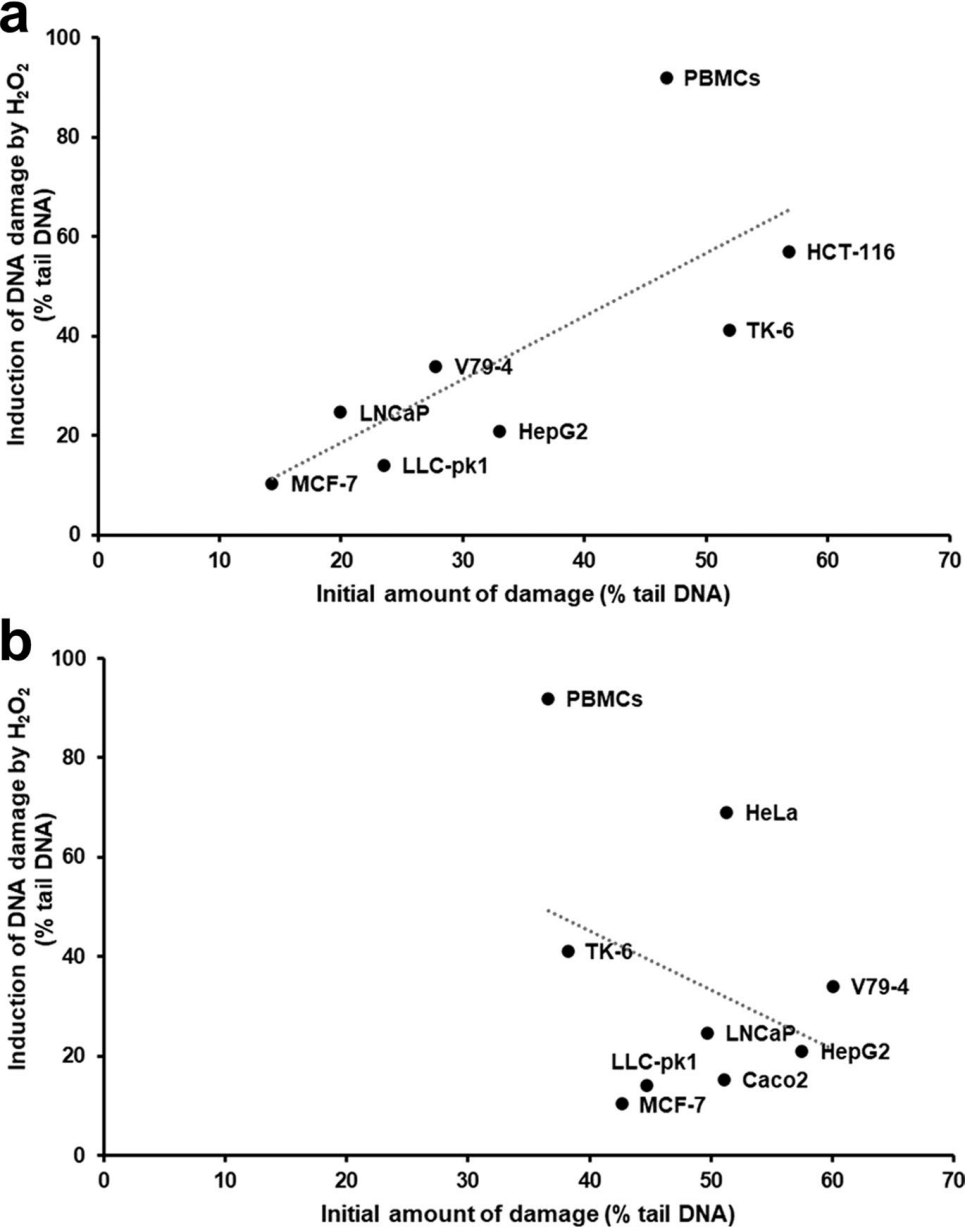
**A** Association between net Fpg-sensitive sites induced by Ro 19–8022 with light in the cellular repair assay and induction of DNA damage by 100 µM H_2_O_2_. **b** Association between net Fpg-sensitive sites induced by KBrO_3_ in the cellular repair assay and induction of DNA damage by 100 µM H_2_O_2_

The antioxidant status of cells was assessed by their resistance to oxidation by H_2_O_2_; a low level of DNA breaks induced by incubation with H_2_O_2_ at 4 °C reflects high antioxidant status. As shown in Fig. 3a, the induction of DNA damage by H_2_O_2_ significantly correlates with the initial level of net Fpg-sensitive sites caused by Ro 19–8022 plus light (*R*^2^ = 0.54, *P* < 0.05)—indicating that the level of net Fpg-sensitive sites induced by Ro 19–8022 is influenced by the cellular antioxidant status. No such association exists between antioxidant status and net Fpg-sensitive sites induced by KBrO_3_ (*R*^2^ = 0.11, *P* > 0.05) (Fig. 3b).

### Timing of repair analyses

**Fig. 4:**
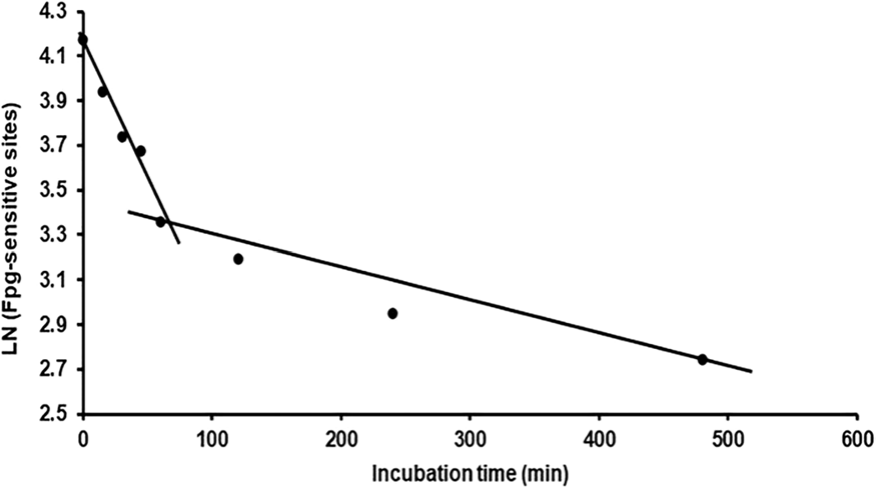
Biphasic removal of Fpg-sensitive sites (LN (Fpg-sensitive sites)) in time (0–480 min). Average of 8 cell lines

The question now arises, how best to express the rate of DNA repair? From a biochemical point of view, the initial rate would be the best measure, but the half-time of removal of damage, *t*_(1/2)_, is another potentially useful parameter (Collins 2014). In an attempt to select the most appropriate measure, we first studied the overall removal of DNA damage by taking the average of all cell lines (Fig. 4). To test if DNA repair is following first-order kinetics, the natural logarithm of DNA damage (average of all cell lines) was plotted versus time, because if this graph is linear and has a negative slope, the reaction is considered to be first-order. Two phases of DNA repair could be distinguished with a linear part in the first 60 min and a linear part in the following period, representing a phase of fast repair and a subsequent period of slow repair (see Fig. 4). On the basis of this observation, *t*_(1/2)_ was calculated for each cell line for 2 different intervals, namely, between 0 and 60 min and between 60 and 480 min (Table 1). On average, the *t*_(1/2)_ was tenfold lower in the first 60 min compared to the period from 60 to 480 min. However, there were large differences between cell types varying between 2.4-fold lower repair in the second phase compared to the first phase in V79-4 cells (*P* = 0.03), to 26.5-fold differences in HeLa cells (*P* = 0.01). Interestingly, a lower repair rate in the first phase was accompanied by a higher repair rate (thus lower *t*_(1/2)_) in the second phase. This may indicate that if more damage remains after 60 min, then higher repair is necessary for the cell to continue the removal of DNA damage. Since this remaining DNA damage at *t* = 60 min cannot be controlled, we suggest that only the interval between 0 and 60 min will reliably reflect DNA repair capacity. Nevertheless, the later phase of DNA repair may still be of interest.

**Table 1:** Half-life of Fpg-sensitive sites after exposure to KBrO_3_ over 2 intervals in various cell types

	Interval 0–60 min (median ± 95% CI)	Interval 60–480 min (median ± 95% CI)	Fold difference	Difference between interval (0–60) and interval (60–480)
V79-4	105.3 ± 13.8	254.1 ± 39.5	2.4	*P* = 0.03
LNCaP	53.4 ± 11.9	538.0 ± 90.2	10.1	*P* < 0.001
LLC-pk1	55.7 ± 19.0	205.0 ± 5.22	3.7	*P* < 0.001
Caco2	65.3 ± 15.8	505.0 ± 103.2	7.7	*P* < 0.001
HeLa	43.1 ± 9.0	1140.7 ± 593.5	26.5	*P* = 0.01
TK-6	39.8 ± 15.9	449.2 ± 297.7	11.3	*P* = 0.035
MCF-7	58.7 ± 15.34	452.6 ± 112.3	7.7	*P* = 0.033
PBMCs	39.9 ± 7.4	374.2 ± 678.1	9.4	*P* = 0.022
HepG2	38.2 ± 27.9	730.7 ± 129.5	19.9	*P* < 0.001

95% CI, 95% confident interval

## Discussion

The cellular repair assay based on the comet assay is a straightforward method for assessing DNA repair activity by measuring the kinetics of DNA damage removal over time. Indeed, Valdiglesias et al. (2020) published an extensive protocol to apply this assay in human biomonitoring by testing differently processed blood samples, including whole blood cells and frozen and fresh PBMCs with different DNA damage-inducing agents. Although this approach has already been applied in human biomonitoring studies, it has not been systematically validated, and various aspects of the assay still need to be addressed before it can be used as a reliable measure of repair rate. Therefore, we further optimized this assay for studying cellular DNA repair kinetics, with a focus on the evaluation of BER, by exposing cells to compounds that induce 8-oxoG, namely, Ro 19–8022 and KBrO_3_. 8-OxoG was subsequently detected using the Fpg-comet assay. The use of Fpg is thought to increase the specificity of the assay for measuring 8-OxoG. However, other types of DNA lesions such as ring-opened purine lesions are also detected, and therefore, the repair kinetics in the current study will not solely reflect the repair of 8-oxoG. The specificity of the assay could be further improved by using the mammalian equivalent of Fpg, 8-oxoguanine DNA glycosylase (human form – hOGG1), and MUTYH glycosylase (Vodicka et al. 2020) for detecting the remaining levels of 8-oxoG with higher precision. Replacing Fpg with other DNA glycosylases would make this assay also fit the study of repair of other types of lesions; for instance, Muruzabal et al. (2020) described the use of hAAG to study the removal of alkylation DNA damage.

Additionally, the type of exposure can improve the specificity; exposure to Ro 19–8022 plus light induces more lesions than just 8-oxoG, and therefore, the repair kinetics reflect the combination of repair by different glycosylases and different types of lesions. Our study showed that exposure to KBrO_3_ resulted in less variation in the level of DNA damage, consistent with conclusions from the recent inter-laboratory ring trial (Møller et al. 2020). Moreover, cell-free experiments indicated that the involvement of hydroxyl radicals and singlet oxygen in the lesions produced by KBrO_3_ can be excluded, and data were consistent with a radical mechanism involving bromine radicals (Ballmaier and Epe 1995). As a result, the net Fpg-sensitive sites induced by KBrO_3_ may be less affected by the antioxidant capacity of cells when compared to Ro 19–8022 plus light. Indeed, in the current study, we showed that the initial level of net Fpg-sensitive sites at T_0_ after exposure to Ro 19–8022 plus light was related to the antioxidant capacity of cells, whereas after exposure to KBrO_3_, it was not (Fig. 2). However, it should be stated here that the damaging properties of KBrO_3_ actually need glutathione (GSH), which is also an important intracellular antioxidant. In this study, we assessed the antioxidant capacity of cells by a short incubation (5 min) with hydrogen peroxide, subsequently measuring the number of strand breaks with the comet assay. This method has previously been successfully used to study the interaction between antioxidant status and genotoxicity (Davies et al. 2001).

Cells have complex molecular mechanisms in response to xenobiotic stress. Redox regulation is an essential mechanism for regulating cellular processes, and there is a balance between ROS (reactive oxygen species) formation and antioxidant defense. Loss of this balance causes excessive DNA oxidation, which could induce DNA repair activity as a “back-up” system. Therefore, as well as studying the association between antioxidant defense and the initial damage induced by 1 µM Ro 19–8022 plus light, we looked at the association between the amount of DNA damage at T_0_ and repair activity. For instance, some cell types (MCF-7 and LncaP) have relatively low initial damage at T_0_ after exposure to Ro 19–8022 plus light (possibly due to a higher antioxidant capacity) and, as a result, showed limited DNA repair activity. These cells may, however, still have the capacity to repair more if more damage had been induced. Therefore, the initial amount of damage seemed to be an important determinant of the DNA repair measurement. In other words, to improve the comparison between cell lines or cells from different individuals in human biomonitoring using the cellular DNA repair assay, it is important to induce similar levels of DNA damage at T_0_. To reach similar initial amounts of DNA damage in the cell lines, we first adjusted doses of Ro 19–8022 plus light (data not shown), but it seemed impossible to achieve this goal. Therefore, another DNA-challenging agent was needed. The ring trial performed by the hCOMET COST Action suggested that KBrO_3_ could replace Ro 19–8022 as a positive control for the Fpg-modified comet assay (Møller et al. 2020a). So far, KBrO_3_ has been used in only a few studies with the Fpg-modified comet assay. TK-6 cells and THP-1 cells were treated with between 1 and 5 mM KBrO_3_ for 1–3 h (Møller et al. 2020; Muruzabal et al. 2020; Platel et al. 2011); Kumar et al. (2022) treated U2OS cells with 0–20 mM KbrO_3_ for 1 h to induce 8-oxoG. The cell viability of U2OS wild type in 10 mM KbrO_3_ is 80%. In our experiments, we exposed cells to slightly higher levels of KBrO_3_ (10 mM), compared to published concentrations, in order to reach similar levels of damage in all cell types.

The direct DNA damage after exposure to KBrO_3_ at T_0_ is assessed after 1 h of incubation with high concentrations of KBrO_3_. In that period, cells may react by upregulating their antioxidant ability, for instance by re-synthesis of GSH or the expression of antioxidant enzymes such as SOD, TrX, and catalase, but DNA damage is induced nonetheless. As a result, the DNA repair pathways can be activated, and the full DNA repair capacity can be studied after 1 h. However, this early induction of DNA repair may interfere with having a reliable measurement at T_0_, because the formation and removal of DNA damage occur simultaneously. Still, after transformation of the data by the natural logarithm, an association was observed that reached linearity, indicating that T_0_ can still be used to reliably calculate the half-life over the period of 0–60 min. After 60 min, the DNA repair activity was observed to slow down, resulting in a larger *t*_(1/2)_. Since the amount of DNA damage at T_60_ can thus not be controlled, we suggest that when comparing cell lines or individuals, only the early phase will reliably reflect inter-individual differences in DNA repair. There have been previous reports of biphasic repair of single-strand breaks (SSBs) (Furuno et al. 1979; Sossou et al. 2005), double-strand breaks (DSBs) (Dolling et al. 1998; Shibata and Jeggo 2020; Stamato et al. 1993; Wlodek and Hittelman 1987; Wu et al. 2002), or both SSBs and DSBs (Olive 1998; V. Calini et al. 2002) induced by ionizing radiation or other genotoxic agents. It is not known at this moment what these two phases represent. There are several possibilities: Some DNA lesions may be more difficult to reach for the DNA repair enzymes since the structure of chromatin could have an important influence on DNA repair kinetics (Wheeler and Wierowski 1983). High levels of DNA damage will result in high repair activity, in agreement with the Law of Mass Action, which states that the rate of a reaction R is equal to the concentration of reactant ([A]) multiplied by a rate of constant (k1), *R* = k1[A]. Therefore, low reactant levels (adduct) will have low reaction (repair) rates and decline as the reactant is used (Kumar et al. 2022; Parlanti et al. 2012).

We have shown significant differences in BER rates among an arbitrarily selected group of cell lines plus a sample of PBMCs. It remains to be seen whether comparable variation exists between PBMCs from different individuals. PBMCs are widely used in human biomonitoring studies, but in theory, other cell types can also be used. PBMCs circulate the whole body and are well-suited for exposure studies in environmental and occupational health research (Esteves et al. 2020; Koppen et al. 2018; Milić et al. 2019). PBMCs are easily accessible and not complicated to handle compared with other cell types. Of course, DNA repair can be different in PBMCs when compared to internal organs. However, while significant correlations were found between DNA repair in organs and PBMCs (Herrera et al. 2009; Slyskova et al. 2012), more work needs to be done in understanding the use of surrogate tissues. Other surrogates used in human biomonitoring include epithelial cells from the eye, tear duct, buccal, or nasal cells to measure DNA damage (Rojas et al. 2014; Russo et al. 2020), but these were not yet used for studying DNA repair.

In conclusion, our study, using the Fpg-modified comet assay to follow the removal of DNA base damage, demonstrates the difference in cellular repair kinetics shown by two different 8-oxoG-inducing agents in various cell lines. Using KBrO_3_ to induce DNA base oxidation resulted in similar initial damage levels in all cell lines, avoiding the complication of varying initial damage apparently owing to differing antioxidant status that is seen with Ro 19–8022. Moreover, *t*_(1/2)_ over the first hour after exposure could be an optimal indicator for measuring the DNA repair ability of 8-oxoG by BER.

### Supplementary Information

Below is the link to the electronic supplementary material.

Supplementary file1 (PDF 208 KB)

## Data Availability

All data generated or analyzed during this study are included in this published article.
