# Supplementary material for: Assay conditions for estimating differences in base excision repair activity with Fpg-modified comet assay
Source: Cell Biol Toxicol. 2023 Mar 17;39(6):2775–86. doi: 10.1007/s10565-023-09801-0 (PMC10693524; doi:10.1007/s10565-023-09801-0)
Supplement: Supplementary file 1 — Supplementary file1 (PDF 208 KB) [file 10565_2023_9801_MOESM1_ESM.pdf]

a

b

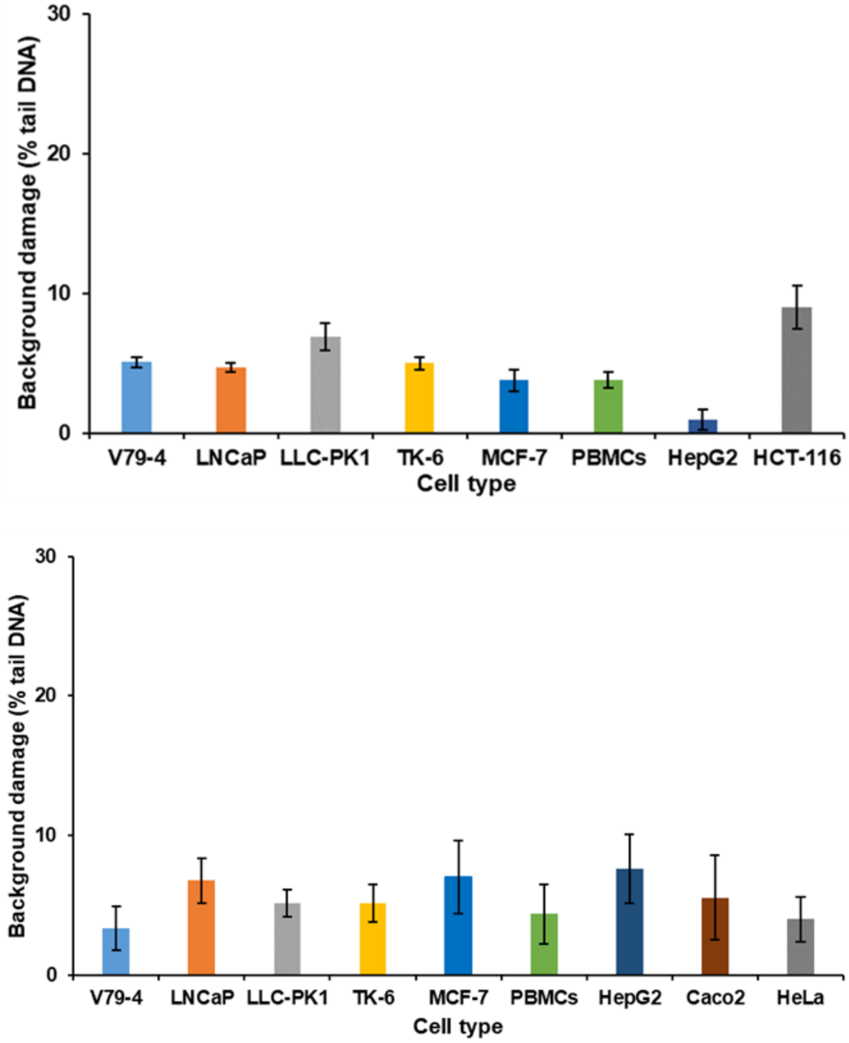


**Fig. S1** Background levels of damage in control cells, not treated with Ro 19-8022+light or $KBrO_3$ in eight cell lines plus PBMCs (a) Ro 19-8022 plus light untreated cells, or (b) $KBrO_3$ untreated cells. These untreated cells are kept in appropriate culture medium at 37 °C for (a) 5 min, or (b) 1 h. Data are shown as mean of median values of three repeat experiments. In the case of PBMCs, there were 2 repeat experiments with each of the 5 samples

**Table S1.** Ro 19-8022 plus light or $KBrO_3$ -induced cytotoxicity determined by a trypan blue exclusion assay

| | Cell viability before exposure (Mean ± SD) | Cell viability after exposure Ro 19-8022 plus light for 5 min (Mean ± SD) $T_0$ | Cell viability after exposure $KBrO_3$ for 1 h (Mean ± SD) $T_0$ |
|---|---|---|---|
| V79-4 | 94.6% ± 3.9% | 93.0% ± 2.5% | 84.3% ± 1.4% |
| LNcaP | 94.2% ± 2.1% | 88.3% ± 2.1% | 84.4% ± 2.1% |
| LLC-PK1 | 94.2% ± 3.0% | 92.0% ± 5.0% | 82.0% ± 1.9% |
| Caco2 | 95.5% ± 1.8% | | 82.8% ± 3.1% |
| HeLa | 95.2% ± 2.7% | | 84.5% ± 1.1% |
| TK-6 | 91.7% ± 6.4% | 89.5% ± 1.5% | 83.8% ± 1.4% |
| MCF-7 | 93.6% ± 5.1% | 94.3% ± 2.0% | 88.6% ± 4.8% |
| PBMCs | 94.1% ± 2.2% | 94.5% ± 1.3% | 85.1% ± 2.0% |
| HepG2 | 95.6% ± 2.4% | 92.0% ± 3.5% | 82.9% ± 5.1% |
| HCL-116 | 96.0% ± 1.3% | 91.4% ± 3.5% | |
